# The significance of intertumor and intratumor heterogeneity in liver cancer

**DOI:** 10.1038/emm.2017.165

**Published:** 2018-01-05

**Authors:** Jinping Liu, Hien Dang, Xin Wei Wang

**Affiliations:** 1 Liver Carcinogenesis Section, Laboratory of Human Carcinogenesis, Center for Cancer Research, National Cancer Institute, Bethesda, MD, USA

## Abstract

Genomic analyses of primary liver cancer samples reveal a complex mutational landscape with vast intertumor and intratumor heterogeneity. Different primary liver tumors and subclones within each tumor display striking molecular and biological variations. Consequently, tumor molecular heterogeneity contributes to drug resistance and tumor relapse following therapy, which poses a substantial obstruction to improving outcomes of patients with liver cancer. There is an urgent need to the compositional and functional understanding of tumor heterogeneity. In this review, we summarize genomic and non-genomic diversities, which include stemness and microenvironmental causes of the functional heterogeneity of the primary liver cancer ecosystem. We discuss the importance and intricacy of intratumor heterogeneity in the context of cancer cell evolution. We also discuss methodologies applicable to determine intratumor heterogeneity and highlight the best-fit patient-derived *in vivo* and *in vitro* models to recapture the functional heterogeneity of primary liver cancer with the aim to improve future therapeutic strategies.

## Introduction

Approximately one million cases of primary liver cancer (PLC) occurs annually and ranks the second most lethal tumor type in the world.^1^ It is predicted that the incidence of PLC will increase by more than 50% by 2030 in the United States.^2, 3, 4^ Notably, in the past 20 years, PLC has been the only cancer with the fastest rising incidence and mortality and with a 5-year survival rate of less than 15% in the United States.^5, 6, 7, 8^ Tumor heterogeneity is the major contributing factor for the refractory nature of PLC to treatment.

Tumor heterogeneity consists of intertumor (tumor by tumor) and intratumor (within a tumor) heterogeneity. Intertumor heterogeneity refers to PLCs from different patients whose altered genotype and phenotype are induced by diverse etiological and environmental factors.^9, 10^ In contrast, intratumor heterogeneity refers to genomic and biological variations within a tumor lesion gained by tumor cell evolution under diverse microenvironments linked to different etiologies. According to the Darwinian evolution selection, a PLC lesion evolves from a single malignant cell into a functionally heterogenous tumor mass with a hierarchically organized tumor cell community, promoting its survival and fitness in response to the various microenvironments.^11^

The most current understanding of PLC heterogeneity is limited to intertumor heterogeneity, mostly focused on molecular subclassification based on genomic profiling. Accordingly, molecular subclassification has identified different patient subtypes according to their genomic profiles with targeted therapy choice. Notably, subclass-related oncogene addiction loops have been identified based on genomic profiling of various tumor subtypes.^12, 13, 14, 15, 16, 17, 18^ This approach has been successfully applied to the management of chronic myeloid leukemia (CML) and breast cancer.^19, 20, 21^ For example, most of CMLs are driven by a fused BCR-ABL oncoprotein, a constitutively activated tyrosine kinase. Ninety-eight percent of BCR-ABL CML responds favorably to the initial treatment of tyrosine kinase inhibitors (TKIs). While BCR-ABL-based subclassification and associated target therapy remain the standard care for CML patients, there is growing evidence showing that a minority of mutational subclones within CML are highly resistant to tyrosine kinase inhibitors,^22, 23, 24^ suggesting that subclassification based on intertumor heterogeneity may not capture the full tumor spectrum. Thus, we need to integrate molecular features of both intertumor and intratumor heterogeneity with functional heterogeneity to improve patient subclassification and response to therapy.

PLC consists of two major histological types,^10^ hepatocellular carcinoma (HCC) and intrahepatic cholangiocarcinoma (ICC), each of which is treated differently, according to their clinical guidelines. Currently, no biomarker-guided targeted therapy is available for HCC or ICC. However, emerging studies aim to HCC or ICC molecular subclassification associated with subtype-related target therapy.^15, 25, 26, 27, 28, 29^ Studies such as whole-genome sequencing (WGS) of HCC reveal frequent mutations in various tumor suppressor genes and oncogenes, including telomerase reverse transcriptase promoter mutations (54–60%), catenin beta 1 (CTNNB1) mutations (11–37%), tumor protein P53 (TP53) mutations (12–48%) and AT-rich interactive domain-containing protein 1A (ARID1A) mutations (4–17%).^30, 31, 32, 33^ However, mutation-specific subtypes are not evident. In contrast, transcriptome-based studies have revealed stable molecular subtypes of HCC and ICC. For example, HCC can be divided into several molecular subtypes based on stemness gene expression patterns.^12, 13, 14, 15, 34, 35^ Similarly, ICC have been categorized to two major subclasses linked to patient outcomes, that is, proliferation subtype and inflammation subtype.^36, 37^ While subclassification based on intertumor heterogeneity is stable among different data sets, improved PLC treatment remains to be seen. Furthermore, much less is known about intratumor molecular heterogeneity of HCC or ICC. It is unclear whether the degree of intratumor heterogeneity is associated with specific tumor types and patient outcomes. Thus, understanding the link between intertumor and intratumor heterogeneity may help improve PLC subclassification and treatment stratification.

## Sources of intratumor heterogeneity

**Figure 1:**
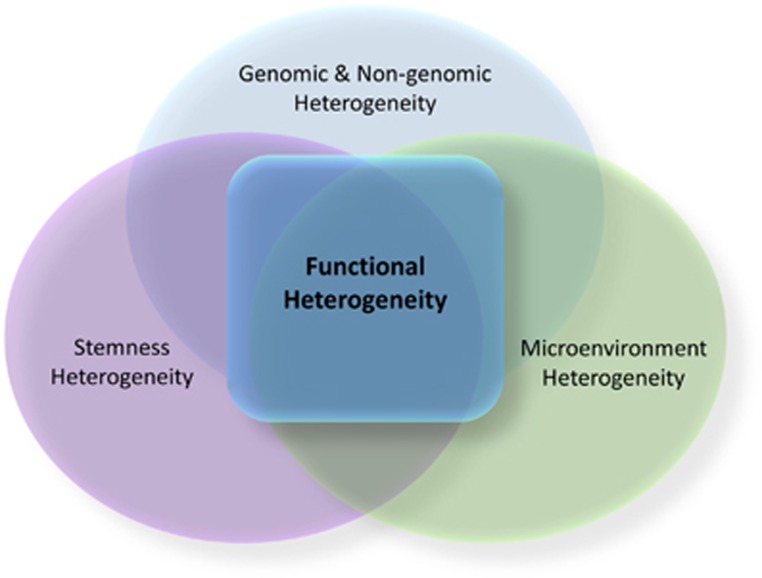
Sources of intratumor heterogeneity of liver cancer. Sources of intratumor heterogeneity of liver cancer include genomic, non-genomic, stemness and microenvironment heterogeneity. Functional heterogeneity refers to abilities of cancer cell populations to undergo cellular proliferation, adaption and drug resistance within a defined microenvironment linked to a specific etiology. These abilities of functional heterogeneity are linked to different sources, including genomic and non-genomic, stemness and microenvironment heterogeneity.

Intratumor heterogeneity has been observed in PLCs in many studies, including histology, ploidy patterns analyses, DNA fingerprinting and WGS.^38, 39, 40, 41, 42^ Here we propose a concept of intratumor ‘functional’ heterogeneity that describes the abilities of cancer cells to undergo cellular proliferation, adaption and drug resistance within a defined microenvironment linked to a specific etiology. We argue that the compositional heterogeneity helps to understand functional heterogeneity, which is key to understanding PLC heterogeneity (Figure 1).

### Intratumor genomic heterogeneity

One important concept about intratumor genomic heterogeneity is the genome-axis evolution model, which describes that gene evolution can increase the survival adaptive function of a cell known as ‘genomic heterogeneity’.^43^ This model suggests that genomic heterogeneity is composed of oncogenes and tumor suppressors that have multiple alterations, including mutation and copy number alterations. Together, these alterations maximize tumor proliferation and survival.^44, 45^ One intriguing example of intratumor genomic heterogeneity and functional heterogeneity is demonstrated in glioblastoma. Accordingly, glioblastoma intratumor with varied epidermal growth factor receptor copy number gain has been measured by Shannon diversity index, demonstrating that functional heterogeneity is linked to increased cell proliferation, resistance to epidermal growth factor receptor inhibitor treatment and aggressive tumor relapse.^46, 47^ Similarly, it has been demonstrated that larger PLC cell populations exhibit greater genomic heterogeneity.^48, 49^ Furthermore, genomic sequencing analyses of HCC specimens have revealed identical mutational profiles of multiregional HCC and ICC and distinct CNVs from single-cell analysis.^50, 51^ However, only a small percentage of mutations were found differently among tumor cell subclones.^42^ While much is known about the heterogeneity for PLCs, the functional consequence of genomic heterogeneity remains unclear.

### Intratumor non-genomic heterogeneity

The dysregulation of the epigenome is one mechanism that contributes to intratumor subclonal variations. Non-genomic intratumor heterogeneity, including histone modifications, DNA hypo- or hyper- methylation, non-coding RNAs, and transcriptional regulators, contribute intratumor heterogeneity by regulating the spatial chromatin organization and altering the transcriptome. Interestingly, aberrant DNA methylation patterns in PLCs are associated with chronic viral hepatitis infections.^52^ Moreover, other studies show that CpG methylation status within the E-cadherin gene promoters is strongly correlated with the heterogeneous expression of E-cadherin in HCC.^53^ Furthermore, promoter hypermethylation of Hexokinase 2 (HK2), an aerobic glycolysis process enzyme gene, has been demonstrated to be associated with poor survival, compared with the hypomethylation of HK2 promoter.^54^ As these studies demonstrate the importance of intertumor heterogeneity in PLCs, more functional studies are required to further understand the association between intratumor non-genomic heterogeneity and its related functions.

### Intratumor heterogeneity and cancer stem cells

Cancer stem cells (CSCs) have been demonstrated to contribute to tumor heterogeneity.^55^ CSCs acquire stem-like characteristics (or stemness) similar to normal stem/progenitor cells, which have been demonstrated to promote liver cancer progression, relapse and intratumor heterogeneity.^35, 56, 57^ Accordingly, Fan’s group recently showed that spatial heterogeneity of CD13+ CSCs is associated with long non-coding RNAs (lncRNAs), including lnc-β-Catm, lncTCF7 and lncBRM, which have been demonstrated to promote the PLC organoid formation.^58, 59, 60^ Although the spatial CSCs heterogeneity has been demonstrated, the functional diverse distribution of CSCs remains unclear.

### Intratumor heterogeneity contributed by tumor microenvironment

Genomic and non-genomic heterogeneity and the presence of stemness from CSCs have provided some degrees of understanding of PLC. Importantly, microenvironment heterogeneity, which can directly interact with tumor cells and influence therapeutic response, has been considered as a novel hallmark of cancer.^61, 62^ Notably, the peritumor microenvironment, from the seed and soil concept, can foster tumor development by reprograming the stromal with the aid of cancer-associated fibroblasts.^63, 64^ *In vivo* studies have demonstrated that tumor cells benefit from the microenvironment, which contributes to promoting cellular diversity by supporting the tumor vasculature and reprogramming immune cell.^62^ Interestingly, tumor-infiltrating lymphocytes can be recruited by HCC cells without secreting the tumor-toxic cytokine interferon gamma (IFN-γ).^65, 66^ These studies suggest that PLC cells could recruit non-toxic infiltrating lymphocytes to promote intratumor heterogeneity and evade from the immune system. HCC studies show that diverse microenvironments are composed of immuno-regulating cytokines, growth factors, immunosuppressors and heterogenous and naturalized stromal cell types. These diverse microenvironments functionally suppress the immune system, by diminishing natural killer cells (NK cells) or enhancing the immunosuppressive regulatory T cells (Treg cells).^67, 68, 69, 70, 71, 72, 73, 74, 75, 76, 77, 78, 79^

### Tumor cell clonal architecture

The process of tumor initiation has not been fully understood, as not much is known about the cellular origin of a tumor mass. In 1976, Nowell^11^ proposed that most tumors arise from a single cell which gains genetic instability within the original clone with sequential selection of branching subclones evolving over time. Notably, recent studies from WGS of different lesions of a tumor mass have extended this model, further suggesting that a tumor cell community is hierarchically organized through a branching or paralleling evolution.^80, 81^ It has been shown that PLC has the propensity to develop multiple subclones, resulting in extended intratumor heterogeneity.^48, 49^ A systematic analysis of PLC intratumor heterogeneity came from a study by Xue *et al.*,^42^ in which the authors performed somatic mutations and copy number variations (CNVs) using a low-depth whole genome and exome sequencing in 43 tumor lesions derived from 10 HBV-positive HCC cases and found evidence of branched evolution linked to HCC. This comprehensive study provides evidence of branched evolution linked to HCC and demonstrate that while tumors from each patient have different clonal hierarchies, satellite nodules share more than 90% of somatic mutations with their primary tumors. Intriguingly, in a patient with multicentric tumors, six lesions show parallel evolution patterns that can be divided into two major subclones, each with its own unique genomic characteristics. In contrast, PLCs with histologically mixed HCC and ICC, satellite nodules shared more than 100 somatic mutations, indicating that subsequent diverse subclones are evident through the genetic evolution of tumor nodules within each PLC. In another study, Ling *et al.*^41^ have analyzed 286 tumor regions from one HCC sample using whole-exome sequencing and genotyping and discovered the evidence of high genetic diversity within a single tumor, indicative of non-Darwinian cell evolution prevalence.^41^ These results suggest that the extent of intratumor heterogeneity varies considerably among patients with HCC.

**Figure 2:**
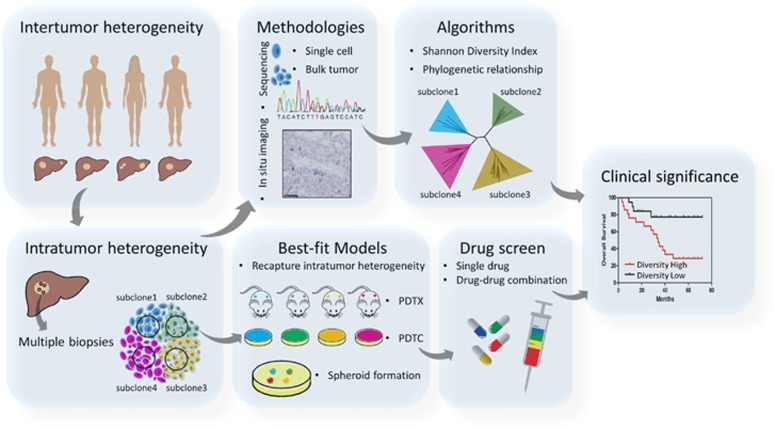
A schematic diagram of understanding, recapturing intratumor heterogeneity of PLC on best-fit models and their applications in drug screen and prognosis. PLC heterogeneity includes intertumor and intratumor heterogeneity. The latter can be dissected by multiple biopsies, which can catch the compositional subclones within each tumor. *In situ* imaging, single cell and bulk tumor sequencing can be performed on the multiple biopsies to help determine the compositional subclones. Intratumor heterogeneity can be quantified by Shannon diversity index and compositional subclones can be categorized by using phylogenetic relationship. *In vivo* PDTX, *in vitro* PDTC and spheroid formation are the preclinically relevant best-fit models, which mostly recapture and preserve the compositional heterogeneity within a tumor and can be used for drug screen. PLC, primary liver cancer; PDTC, patient-derived tumor cell; PDTX, patient-derived tumor xenograft.

To address the extent of PLC heterogeneity, multiple biopsies from a single tumor is required. This can be accomplished by collecting multiple biopsies from different geographical regions within a tumor or patient. Such analyses will reveal tumor cell hierarchies at the genomic and non-genomic level, including the presence of CSCs and the diversities within the tumor microenvironment at a given time (Figure 2).^80^ In addition, longitudinal sampling from each patient including prior and post treatment can be implemented.^44^ This approach improves our understanding of the PLC evolution during tumor progression. Notably, some has suggested that the use of imaging techniques is sufficient for PLC diagnosis. Given the extent of genomic and biological heterogeneity associated with PLC, such a view is shortsighted.^82^ Therefore, a comprehensive and systematic analysis of multiple biopsies is recommended to uncover the PLC functional heterogeneity that drives tumor evolution.

## Approaches to study intratumor heterogeneity

Several studies in recent years have begun to explore methodologies to study intratumor heterogeneity. Here we outline some of the current methodologies that have been successfully used to characterize intratumor heterogeneity (Figure 2).

### Intratumor heterogeneity from genome-wide studies

Morphological intratumor heterogeneity has long been observed by pathologists. However, intratumor molecular heterogeneity has only been appreciated in recent years due to WGS technologies. The first study to address intratumor molecular heterogeneity was based on multiregional sequencing of renal carcinomas and associated metastatic sites.^80^ Mutational intratumor heterogeneity was observed for multiple tumor suppressor genes and further analysis of multiple regions of PLCs provide evidence of the trunk and branch mutational profile.^41, 42^ Moreover, Zhang’s group studied HCC heterogeneity using allelic frequency profiles of frequently mutated genes across genomes and exomes based on deep sequencing of two separate biopsies from each tumor.^83^ Among 42 HCC cases analyzed, they found evidence of diverse modes of genomic alteration in HCC. These studies suggest that given the extent of intratumor mutational heterogeneity, multiple sampling of only a few regions to determine driver mutations may not necessarily be adequate to capture the true mutational landscape of a tumor type.

### Intratumor heterogeneity at single-cell level

The recent development of single-cell genome sequencing technologies has generated many new insights into complex biological systems including human cancer.^84^ Single-cell analysis may provide the level of sensitivity and specificity to study tumor cell diversity in a tumor cell ecosystem.^85, 86, 87^ One latest technological advance includes the development of scTrio-seq, a single cell triple omics method developed by Hou *et al.*^51^ to simultaneously analyze genome (CNVs), DNA methylome and transcriptome in a single cell. While single-cell technology is useful to study tumor cell diversity in each tumor cell ecosystem, it lacks information about topological space within a tumor cell community. New approaches such as single-cell gene expression profiling with multiplexed error-robust fluorescence *in situ* hybridization may help resolving this issue.^88, 89^ In addition, while single-cell RNA transcriptome has provided sufficient resolution to distinguish single cells, whole genome or exosome sequencing technologies are not sufficient to provide a comprehensive view of global genomic landscape of a tumor at the single-cell level. Therefore, identifying cancer driver genes linked to single tumor cells is a challenge.

### Circulating tumor cells

Circulating tumor cells (CTCs), found in blood and originated from aggressive subclones of the primary tumor, are responsible for metastasis and tumor relapse. A meta-analysis of HCC CTC studies revealed that CTCs are positively correlated with poor prognosis.^90^ However, not all CTCs are tumorigenic, suggesting that these cells are heterogeneous.^91, 92, 93^ Molecular analysis of CTCs may provide information on tumor cell heterogeneity. A deep understanding of CTC heterogeneity may facilitate our ability to identify key molecular targets to improve cancer therapy. Interestingly, Miyamoto *et al.*^94^ demonstrated considerable heterogeneity among CTCs by analyzing 77 intact CTCs from 13 prostate cancer patients by single-cell RNA sequencing. As CTCs are rare, therefore methods that can accurately detect and isolate them for downstream analysis are crucial. In a study by Lohr *et al.*,^95^ the authors developed a microscope-based technology to detect and isolate CTCs from multiple myeloma (MM) patients for single-cell RNA-sequencing analysis. They found that CTCs provide the same genetic information as bone marrow multiple myeloma cells, providing a more clear overview of the tumor itself than bone marrow biopsies thus enabling the classification of MM to improve precision medicine.^95^ In addition, Kalinich *et al.*^96^ developed a high-throughput microfluidic CTC-iChip, which can be used to enrich HCC CTC while excluding hematopoietic cells from the blood. It will be interesting in determining if these methods allow assessment of CTC heterogeneity. Moreover, whether these methods can be incorporated into clinical practice, especially PLC management, remains to be determined.

### Spatial organization of a tumor cell community

*In situ* methods are desirable techniques for understanding PLC heterogeneity because it can address the spatial organization of a tumor cell community. This method has been used to detect DNA and RNA using formalin-fixed paraffin-embedded tissue microarrays in a high-throughput manner.^97, 98^ With the use of high-resolution microscopy, CNVs, genetic mutation profile and RNA quantification can be profiled at the single-cell level. This is evident in a study by Kimura *et al.*^99^ who demonstrated the presence of molecular heterogeneity by *in situ* hybridization with a chromosome 17-specific DNA probe. The authors found that distinct subclones in 2/25 HCC samples with chromosome 17 copy number alterations and ploidy patterns.^99^ Others have also determined topological features of intratumor subclones by *in situ* approaches.^44^ While the current *in situ* hybridization method still has a poor resolution as only a few molecular features can be assessed at one time. More sensitive methods with higher densities of DNA/RNA probes are being developed to help further our understanding of spatial organization of a tumor subclone.^89^ Additional mathematical models such as the Shannon diversity index method are being explored to precisely measure the degree of intratumor heterogeneity in a tumor cell ecosystem. New models to incorporate spatial organization with Shannon diversity index should be exploited to measure the true dynamic of a tumor cell community.

### Preclinically relevant best-fit models to study intratumor heterogeneity

Experimental models are very useful tools to explore molecular mechanisms that drive tumorigenesis, and ideally used to study intratumor heterogeneity. Appropriate models that mimic human diseases are necessary to provide the clinical relevance.^100, 101^ Among various available models, patient-derived tumor xenografts (PDTXs) and matched patient-derived tumor cells (PDTCs) have been the preferable choices. Accordingly, Bruna *et al.*^102^ have generated a living biobank of human breast cancers in order to adequately capture the inter- and intratumor heterogeneity in pre-clinical models. Remarkably, the intratumor genomic clonal architecture of the originating tumors is largely preserved in PDTXs and matched PDTCs.^102^ Using this resource coupled with a high-throughput response and resistance screens, they have successfully assessed drug responses in these models.^102^ In addition, *in vivo* PDTX can recapture up to 82.5% of *ex vivo* PDTCs in terms of the drug response. These results suggest that both PDTC and PDTX represent a powerful resource for pre-clinical pharmacogenomic studies. Most recently, Gao *et al.*^103^ also observed considerable intratumor heterogeneity and branched evolution in HCC samples that correspond to existing pharmacologic agents using PDTCs. These studies indicate that PDTX and PDTC coupled with the use of their genomic information are useful platforms for pharmacologic assessments linked to intratumor heterogeneity. Thus, improving assessment of the complexity of PLC intratumor heterogeneity will provide a better understanding of functional heterogeneity, which may be linked to therapeutic responses.

## Future perspective

Our understanding of PLC heterogeneity has been improved in recent years, but many challenges are still ahead such as a precision measurement to recapture and preserve the diversity of a tumor cell community linked to patient outcomes including treatment response. The PLC ecosystem is dynamically changing during therapy as resistant clones may be selected and expanded throughout tumor evolution. To improve HCC therapies, understanding the impact of tumor heterogeneity must be appreciated. First, we need to understand the diversity of a given tumor cell community and to learn their collective behaviors. By measuring the phylogenetic relationship among different cells and using the Shannon diversity index through subclone bulk or single-cell sequencing (Figure 2), we may understand the complexity of a tumor cell community and determine the degree of diversity among individual patients. Second, to further understand the pattern of intratumor molecular heterogeneity of a given tumor and its relationship to other tumor types, we need to better understand the dynamics of the molecular subtypes of PLC linked to ethnicity, etiological factors and patient outcomes. A well-defined patient data ecosystem including diverse patient populations is vital step to achieve this goal.^82, 104^ Third, given the importance of the molecular and phenotypical intratumor heterogeneity of PLCs, the access to a living biobank of PLCs will help build the much-needed database to further understand features of intratumor heterogeneity. Furthermore, a drug response database from high throughput single drug and drug–drug combination screen using *in vivo* PDTX and *in vitro* PDTC models will prove effective for developing targeted therapies (Figure 2). Together, these efforts will improve PLC preclinical trial design, consequently, improving our discovery of new and effective therapeutics for PLC. Finally, a better understanding of the microenvironment diversity, and the dynamic interplay between the microenvironment and tumor ecosystem, will help improve our understanding of a patient’s response to immune therapy or molecularly targeted therapies.^105^ With the development of better molecular technologies, we may be able to ultimately find solutions to improve outcomes of patients who suffer from this dreadful disease once for all.

## Publisher’s note

Springer Nature remains neutral with regard to jurisdictional claims in published maps and institutional affiliations.
